# Single-Pass VDD Pacing Lead for Cardiac Resynchronization Therapy: A Reliable Alternative

**DOI:** 10.3390/mi12080978

**Published:** 2021-08-18

**Authors:** Silvius-Alexandru Pescariu, Raluca Şoşdean, Bogdan Enache, Răzvan I. Macarie, Mariana Tudoran, Cristina Tudoran, Cristian Mornoş, Adina Ionac, Sorin Pescariu

**Affiliations:** 1 Department VI, Discipline of Cardiology, University of Medicine and Pharmacy “Victor Babes” Timisoara, E. Murgu Square, Nr. 2, 300041 Timisoara, Romania; pescariu.alexandru@umft.ro (S.-A.P.); enache.bogdan@umft.ro (B.E.); mornos.cristian@umft.ro (C.M.); ionac.adina@umft.ro (A.I.); pescariu.sorin@umft.ro (S.P.); 2 Cardiology Clinic, Institute of Cardiovascular Medicine Timisoara, 300310 Timisoara, Romania; macarie.razvan@umft.ro; 3 Research Center for Cardiovascular Diseases, Institute of Cardiovascular Diseases, 300310 Timisoara, Romania; 4 Department VII, Internal Medicine II, Discipline of Cardiology, University of Medicine and Pharmacy “Victor Babes” Timisoara, E. Murgu Square, Nr. 2, 300041 Timisoara, Romania; tudoran.mariana@umft.ro; 5 Center of Molecular Research in Nephrology and Vascular Disease, Faculty of Medicine, University of Medicine and Pharmacy “Victor Babes” Timisoara, E. Murgu Square, Nr. 2, 300041 Timisoara, Romania; 6 County Emergency Hospital, L. Rebreanu Str., Nr. 156, 300041 Timisoara, Romania

**Keywords:** cardiac resynchronization therapy (CRT), VDD–CRT system, complication rate, atrial undersensing, VVI pacing

## Abstract

(1) Background: Cardiac resynchronization therapy (CRT) systems can be simplified by excluding the atrial lead and using a Ventricular-Dual-Dual (VDD) pacing lead. Possible disadvantages might include atrial undersensing and Ventricular-Ventricular-Inhibition (VVI) pacing. Because literature data concerning these systems are scarce, we analyzed their benefits and technical safety. (2) Methods: this retrospective study compared 50 patients implanted with VDD–CRT systems (group A), mainly because of unfavorable venous anatomy concerning the complication rate, with 103 subjects with Dual-Dual-Dual (DDD)–CRT systems (group B) implanted during 2000–2016 and 49 (group C) during 2016–2020. To analyze the functional parameters of the devices, we selected subgroups of 27 patients (subgroup A) and 47 (subgroup B) patients with VDD–CRT in 2000–2016, and 36 subjects (subgroup C) with DDD–CRT implanted were selected in 2017–2020. (3) Results: There was a trend of a lower complication rate with VDD–CRT systems, especially concerning infections during 2000–2016 (*p* = 0.0048), but similar results were obtained after rigorous selection of patients and employment of an upgraded design of devices/leads. With a proper device programing, CRT pacing had similar results, atrial undersensing being minimal (*p* = 0.65). For VDD-systems, VVI pacing was recorded only 1.7 ± 2.24% of the time. (4) Conclusions: In patients with a less favorable venous anatomy, VDD–CRT systems may represent a safe alternative regarding complications rates and functional parameters.

## 1. Introduction

The rate of electronic cardiac device implantation, including cardiac resynchronization therapy (CRT) devices, is rising continuously as a result of an increase of the medium life expectancy and in the aging population and also because of the widening of the indication range [1,2,3]. However, studies have shown a disproportionate increase in device-related complications, especially concerning infections. This is mostly due to the high complexity of the implanted systems [4,5]. CRT systems are among the most complex, with a high number of leads and a laborious, time-consuming implant intervention. Unlike traditional pacing systems which require a maximum of two leads inserted into the heart’s cavities (one in the right ventricle and one in the right atrium), CRT systems add a lead in the coronary sinus, adjacent to the left ventricle. This procedure hopes to adjust the electrical vectors of the heart muscles, the result being a better contraction of the myocardium. However, these sorts of intracardiac devices were proven to have a higher complication rate, considering both infections and lead-related issues, compared to the simple single-chamber and/or dual-chamber systems [6,7]. This is a serious problem, as these complications have a serious impact on the patient’s health, quality of life, and even mortality. They also lead to a serious increase in therapy costs, because of the need for reintervention and sometimes replacement of the entire system.

A simplification of these systems, e.g., decreasing the number of leads by using a single-pass ventricular-dual-dual (VDD) electrode lead to replace the right atrial and right ventricular (RV) lead, could be beneficial by excluding the atrial lead from the pacing system, hence rendering the procedure simpler with a less foreign body within the cavities. Functional VDD pacing mode (Dual-Dual-Dual (DDD) pacing mode with a low programmed minimal heart rate) is the most widely used procedure in the representative CRT clinical trials, as atrial sensing was proved to be beneficial by preserving the atrial synchrony compared to atrial pacing. Furthermore, several reports in the literature even suggested the use of single-pass VDD pacing leads, for CRT, to be beneficial in terms of costs, intervention, and fluoroscopy times and also in terms of the complication rate, but these reports are based on very small numbers of patients [8,9,10]. These possible benefits should be studied though, as VDD CRT systems, even if possessing only two leads, cannot be considered similar to simple dual-chamber systems. A longer implantation time is required, usually because of the coronary sinus lead positioning, and the patients usually have a different and higher number of comorbidities. Concerning simple dual-chamber pacemakers implanted for atrioventricular block (AVB), single-lead VDD systems are currently considered and proved to be a good alternative to DDD systems when implanted in centers with experience in this regard, even accounting for the possible atrial undersensing by the atrial floating electrode on the VDD lead. Previous studies proved minimal atrial sensing problems with these systems when respecting the best-suited implantation and programming criteria [11,12,13].

However, there are no consistent data on the efficacy of VDD leads in properly maintaining CRT and in inducing the expected effects on patients’ evolution. CRT patients have a different pathology and an increased number of comorbidities compared with patients with AVB. Dilated cardiomyopathy for which CRT is implemented and also advanced age are cited as possible factors that negatively impact atrial sensing [14], and a significant number of patients receiving CRT are part of the aging population. For CRT, it is very important to have good atrial sensing, as atrial undersensing would lead to a lack of biventricular pacing and loss of resynchronization, and it was proved that good results for symptoms and survival were obtained with CRT pacing higher than 93% and even better than 98% [15,16]. Further, a decrease in the patient’s heart rate below the minimum programmed heart rate would lead to Ventricular-Ventricular-Inhibition (VVI) pacing because of the theoretical incapacity of the floating dipole to pace the atrium, with loss of atrioventricular (AV) synchrony. This may negatively impact the patients’ evolution.

Thus, in this study, we aim to analyze the possible benefits of implementing CRT through the simplified VDD CRT pacing system in terms of complications necessitating a new intervention and to verify the competence of these systems in terms of CRT maintenance and one-year mortality, as compared with DDD CRT systems. 

## 2. Materials and Methods

### 2.1. Study Population

In the first stage, a single-center cohort of 153 patients consecutively implanted with a CRT pacing system (VDD and DDD type) at the Institute of Cardiovascular Diseases from Timisoara, Romania, from 2000 to 2016, with a minimum follow-up period of one year (except for the patients that experienced death before this time threshold), was included in the study. The patients were divided into two groups: group A consisting of 50 subjects implanted with VDD CRT pacing systems, and group B including 103, patients implanted with DDD CRT pacing systems. During the aforementioned time period, some of the pacing leads used were thicker (7 French) and less flexible due to the polymer composite of the coating; thus, excluding one of the three leads in CRT devices by using a VDD system seemed like the natural choice. The two groups of patients were comparatively analyzed for complication burden, specifically for device infection/exteriorization and lead-related problems to verify the possible benefits of the two lead CRT systems. In the second stage, starting in 2017, the usage of VDD pacing systems rapidly declined due to the introduction and availability of thinner (6 French), more flexible, and more biocompatible leads. According to the recommendations of the of the European and American Society for Cardiac Pacing and Resynchronization and of the European Society of Cardiology for the treatment of Heart Failure [17,18,19,20], and after reconsidering the protocols and rigorously analyzing the indications and benefits of VDD CRT pacing systems, in 49 patients upgraded DDD CRT pacing systems with thinner leads were implanted until January 2020 when the study was seriously impacted by the outbreak of the coronavirus disease pandemic of 2019 (COVID-19). Taking into consideration the improved hardware largely available, modern pacing leads carry a lower risk of complications. These patients represented group C and their clinical data, evolution, complications, and mortality rates, as well as device functionality-related data were analyzed and compared to the results obtained in the previous two groups.

For the analysis of technical and functional properties of the two types of CRT devices, subgroups of patients with all available data concerning the timely interrogation and functional parameters of the devices, while in sinus rhythm, during at least one year of follow up were selected from each group. The three subgroups were comparatively analyzed for acute and chronic pacing system functional parameters, specifically for right atrial sensing, the percentage of different pacing modalities (CRT, biventricular DDD, biventricular VVI), and ventricular sensing, to verify the efficiency of the two lead CRT systems.

All patients signed at the admission in the hospital the standardized informed consent form required by the national health system of our country, by which they consented to their data being used for research and medical education purposes.

### 2.2. Clinical and Echocardiographic Data Collection

The baseline clinical and echocardiographic variables that could have influenced the outcomes were retrospectively collected from the patients’ files stored in the hospital’s archive and from the electronic data-base of the hospital. Only the left ventricular (LV) ejection fraction values determined by the Simpson method were taken into consideration. Data on patients’ deaths outside the hospital were collected from their families or from the general practitioners.

### 2.3. CRT Systems’ Implantation and Programing

All CRT systems were implanted according to the indications of current guidelines from the joint task force of the North American Society of Pacing and Electrophysiology, the American College of Cardiology, the American Heart Association, and the Working Groups on Arrhythmias and Cardiac Pacing of the European Society of Cardiology (NASPE/ACC/AHA/ESC) for pacing and CRT [1,3,4,17,18,19,20], as well as of the Clinic’s protocols. Several distinct specifications regarding VDD CRT pacing systems, as patients’ characteristics, pacing systems, and implantation modality were carefully selected to ensure the best effect. VDD CRT pacing systems were usually implanted in patients with difficult anatomic conditions (e.g., particular constitutional characteristics, difficult venous approach necessitating multiple punctures, low quality/fragile venous system, etc.), with a left atrium diameter of less than 5 cm, a non-severely dilated right atrium, with normal left atrial function, specifically without sinus node disease (because of the theoretical lack of atrial pacing capability), and without documented atrial tachyarrhythmia episodes (because of the possible remission of these paroxysmal episodes by atrial pacing). The atrial dipole was placed in the middle or the upper part of the right atrium since this position was proved to ensure the best detection and stability, aiming for an acute atrial sensing threshold of at least 1–1.5 mV. The VDD leads were selected to have an inter-electrode distance of 15 cm (as a high distance is best suited for dilated cardiomyopathy patients), and an atrial dipole of 3 cm (as it can ensure optimal atrial sensing and avoids far-field ventricular sensing) [11,13,21,22]. The minimal pacing heart rate was programmed at 40–45 bpm, and the atrial sensitivity was set to the maximal values. For DDD systems, the minimal pacing heart rate was set to 45–50 bpm. All patients received three doses of an antibiotic (usually amoxicillin with clavulanate) and the control visits were set at 1 month, 3 months, and every 6 months thereafter.

### 2.4. Device and Intervention Related Data Collection

Baseline device and procedural related variables that could influence the outcomes and also data on complications that needed reintervention- pocket and/or lead-related were retrospectively collected from the procedural logbooks stored in the hospital’s electrophysiology laboratory archive. We considered a complication to be acute when it occurred in the first month after implantation and chronic when it occurred outside this period. Data on chronic pacing system functional parameters were collected retrospectively from the specific device programmer memory and printed device diagnostics. For some patients, these were collected prospectively by device interrogation, when coming for the routine visit, or another hospital admission, after signing an informed consent document. For most of the patients, chronic CRT parameters were evaluated after at least one year.

Concerning the percentage of CRT, DDD pacing, and ventricular sensing, these were calculated and represented exactly by the device’s software. We collected data on CRT pacing also as percentage intervals, taking into account the reference values of two very large trials, concerning the impact on response and survival (<93%, 93–98%, >98%) [15,16]. Further, based on the same principle, we collected data on ventricular sensing when encountered in more than 2%. This parameter offers indirect data on atrial undersensing as it comprises the patient’s non-paced QRS complexes resulted from lack of atrial sensing (undersensing in normal conditions and/or atrial tachyarrhythmia) with a consecutive lack of biventricular pacing, but also ventricular premature beats, that may not always be possible to verify with an intracardiac ECG recording from the devices’ memory. The percentage of VVI pacing was considered to be equal to the percentage of DDD pacing in VDD systems programmed to pace in DDD mode (as VDD systems are theoretically incapable of atrial pacing). For VDD systems programmed to pace in VDD mode, it was approximated from the rate histograms, considering VVI pacing when the atrial rate decreased below the minimal programmed heart rate. As VVI pacing could not be measured exactly all the time, we decided to collect these data also as percentage intervals, besides the absolute values. Chronic atrial sensing values were retrospectively collected from the device memory or prospectively by performing the atrial sensing test.

Only the device function interrogated at periods of at least three months were considered. Further, whenever possible, the sessions analyzing the period immediately previous to clinical and echocardiographic reevaluation were considered. The sessions recorded when a lead complication was detected were excluded.

The study was approved by the Institutions’ Ethics Committee of the University of Medicine and Pharmacy “Victor Babes” Timisoara, Nr 1/2012.

### 2.5. Statistical Analysis

Statistical analysis was performed by using SPSS v.25.0 (Statistical Package for the Social Sciences, Chicago, IL, USA) for Linux Mint 19 (The Linux Foundation, San Francisco, CA, USA). Continuous variables were presented as the mean and standard deviation (SD) or median and associated quartiles (Q1-25 percentage quartile, Q3-75 percentage quartile), and categorical data were presented as counts (percentages). The bias-corrected and accelerated bootstrap interval (1000 bootstrap samples) was used to calculate the 95% confidence interval. Descriptive and inferential statistics analysis was employed to characterize the study population. The results of the Shapiro-Wilk normality test showed a non-Gaussian distribution, which is why we continued to use nonparametric tests. To evaluate the prevalence of various characteristics in study groups, we applied the chi-squared test (χ^2^) and Fisher exact test (Freeman-Halton extension). For comparing the three groups (A, B, C), we employed the Kruskal–Wallis test, followed by post-hoc analysis with the Mann–Whitney U test and Bonferroni correction for pairwise comparisons. A *p* value of less than 0.05 was considered to indicate a statistical significance.

## 3. Results

There were a total number of 50 patients receiving VDD CRT pacing systems (group A), 103 subjects receiving DDD CRT pacing systems during 2000–2016 (group B), and 49 patients implanted during 2017–2020 benefiting from the same type of stimulation (group C). The groups were similar considering patient, device and intervention-related characteristics that could influence the study outcomes (Table 1).

The follow-up periods were similar for all groups: 64.74 ± 47.84 months and 50.59 ± 31.27 month for VDD systems and 24.6 months for DDD systems, (*p* = 0.06).

**Figure 1:**
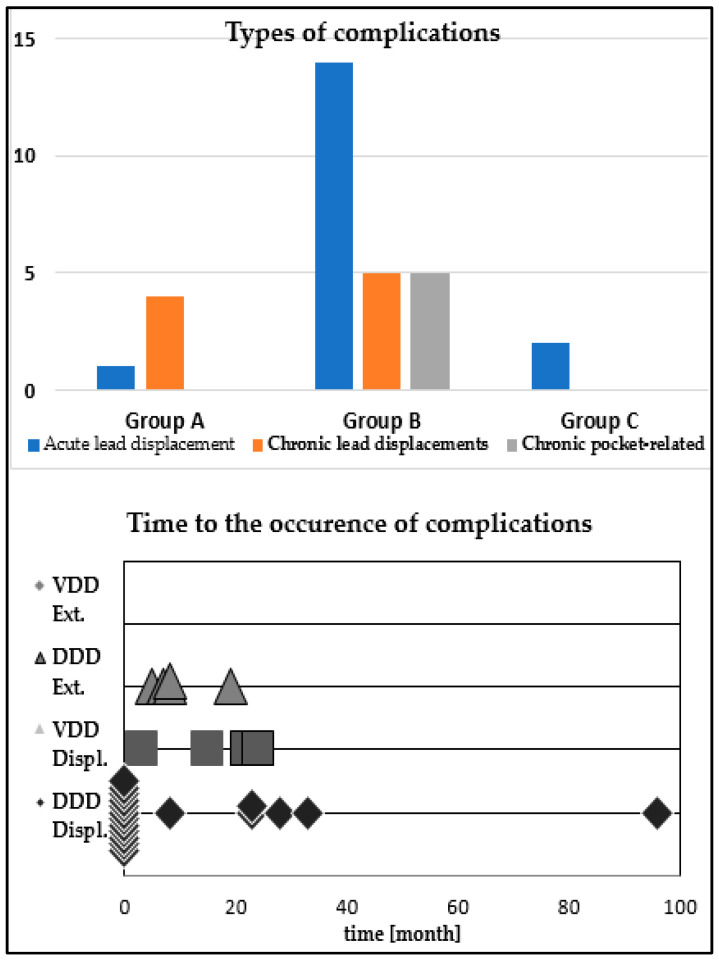
Graphic representation of the prevalence and time to diagnosis of each type of complication in the three study groups.

We encountered a total of five complications (10%) in group A versus 24 (23.3%) in group B and two complications (4.1%) in group C. In group A, there were only lead-related complications: one (2%) in the first month, three chronic (between 3 and 12 months) displacements of the coronary sinus lead, with an associated fracture in one, and two chronic VDD lead displacements, with no pocket related complications; in group B there were 19 (18.44%) lead-related complications: 10 acute and three chronic displacements of the coronary sinus lead, two chronic displacements of the right ventricular lead, and four acute displacements of the right atrial lead (*p* = 0.23), and five chronic pocket-related complications: three device exteriorizations without proven infection and two with proven infection (*p* = 0.17); in group C, there were only two acute lead displacements of the coronary sinus lead. The prevalence and timing of these complications are represented in Figure 1 and Table 2.

The 1-year all-cause mortality was 8% in the VDD group (4 out of 50 patients), 9.7% in the DDD group (10 out of 103 patients), and 6.1% in group C (3 subjects), but a possible more advanced underlying disease in the DDD systems’ groups must be considered, even if the left ventricular ejection fraction (LVEF) and New York Heart Association (NYHA) classes were similar. In most cases, VDD systems were implanted in patients without atrial tachyarrhythmias and or associated sinus node disease. No death was related to the reported complications.

In order to test the efficacy of the VDD CRT pacing systems, 27 patients of group A, 47 of group B, and 36 of group C fulfilled all requirements to be included in the study subgroups. Clinical, echocardiographic, and therapeutic variables that could influence the study outcomes were similar for both subgroups (Table 3).

Pacing and sensing variables are shown in Table 3. As expected, there were significant differences between the three subgroups concerning the percentage of DDD and VVI pacing modes. DDD pacing was detected in subgroups B and C. For the VDD CRT systems, even if programmed DDD and some of them were capable of atrial pacing, the pacing threshold was much higher than the programmed one. The unwanted VVI pacing was detected in a small percentage in the study subgroup.

The acute atrial sensing, by respecting the criteria of correct implantation, was similar to the atrial floating electrode and the fixed atrial electrode. Although the atrial floating electrode is known to have a high sensing variability with time and position, by setting the sensitivity in the highest range, there were no significant undersensing events detected. The percentage of CRT pacing was similar between the two systems. We considered it more accurate and representative to evaluate this parameter instead of chronic atrial sensing, given the variability of the second parameter. Further, a percentage of CRT pacing as high as possible is the main functioning goal of any CRT system.

## 4. Discussion

Our results show that there is a substantial trend of the lowering complication rate by implementing CRT through the less complex, two-lead VDD systems, a trend at the limit of being statistically significant. The difference was significant when considering infectious complication rates separately, as in our patients, no such problems occurred with the VDD systems. These benefits came with a good functioning and patient evolution, similar to that of the standardized DDD CRT systems, without an increase in the non-responder rate compared to these systems. VVI pacing could not be completely avoided, but there was a very low percentage. The atrial floating electrode did not have any significant impact on CRT pacing, as it seems it did not reach the limit of atrial undersensing in a significant amount of time. A significant reduction of complications was also achieved after a rigorous review of the department’s protocols, an adequate selection of patients with less severe heart failure (even in NYHA class II), and by employing updated devices with thinner, more flexible leads.

Concerning the complication rate, our results are similar to the ones of the FOLLOWPACE trial, one of the most recent and large prospective trials, conducted on 1517 patients from 23 implant centers, even though it included only simple devices. The authors found a complication rate of 15.6% during the first year, 18.3% in the first three years, and 19.7% in the first five years [23]. We found in our study a global rate of 11.41% in the first year and 15.34% during the entire follow-up (55.08± 35.67 months). Further, a large retrospective study including a number of 28 860 patients from the Danish Pacemaker Registry between 1997 and 2008 found in both simple and CRT systems, 2.3% right atrial (RA) lead displacements, 2.2% RV lead displacements, and 4.3% LV lead displacements [7]. Globally, in the first month, we found a similar percent of 9.15% total lead displacements, with a very low percent in VDD CRT systems, of 2%. One of the most important meta-analyses, evaluating only biventricular systems, including 25 well-known CRT trials, with a total number of 9082 patients, also revealed a peri- and postimplant complication rate of 1.4% for infectious complications and 6.2% for lead-related complications. The included studies had different complication follow-up periods but mostly between one and 12 months [24].

High negative variability of chronic atrial sensing with the floating atrial dipole was often described with simple VDD systems. Shurrab et al. [21] performed a meta-analysis including eight studies conducted between 1998 and 2012 with a total number of 1942 patients, 922 implanted with simple VDD systems, and the rest with simple DDD systems for high degree AVB. Besides the benefits regarding complications and intervention and fluoroscopy times and the lower incidence of atrial fibrillation, they also found a higher variability of the chronic atrial sensing with VDD systems. Globally, this was significantly reduced as compared to DDD systems, but when considering only the values from the end of the follow-up period, the differences, although detectable, lacked statistical significance. They also found atrial undersensing in a significantly higher percent with VDD systems (10.6% versus 3.6%). In our study, ventricular detection including non-paced QRS complexes resulting from atrial undersensing was detected in a similar percent with the two CRT systems. Atrial undersensing could not be exactly estimated, as it does not result in VVI pacing as with simple devices, but the low percent of ventricular detection is a good outcome. Schaer et al. [12] conducted a study including 320 patients implanted with simple VDD systems for the same reason, adopting a similar implant procedure and device programing as in our study. They also found a very low percent of VDD pacing failure, of 5%, from which 4% was due to atrial undersensing, and 2% needed a system upgrade. No VDD system in our study needed an up-grade to a DDD CRT system, but we also had two VDD lead problems (4%) necessitating intervention compared with four atrial lead problems (3.88%) in the DDD systems, lacking statistical significance. Marchandise et al. [22] also proved atrial sensing to be significantly reduced with the atrial floating dipole (1.0 ± 0.8 mV versus 2.5 ± 1.8 mV), with a weak atrial sensing in 19.1% and atrial undersensing in 7.1% of VDD patients versus 0.4% DDD patients. In this study though, atrial sensitivity was not programmed to its highest possible values. Atrioventricular synchrony was maintained over 95% of the time after sensitivity adjustment, and reintervention necessity for this issue was not significantly different between the two systems. The relatively good atrial sensing with the VDD lead, even in patients with dilated cardiomyopathy and reduced ejection fraction [23,24] factors that were demonstrated to have a negative impact on this parameter, is most probably due to the very rigorous patient and lead selection, and a very strict implant procedure and device programming, in order to increase as much as possible, the detected P wave’s amplitude with these systems.

Even if they proved to be a good alternative for DDD systems, simple VDD systems are rarely used currently because the studies lack consistent results regarding their beneficial effects on complications and also due to the routine and ease of implanting simple DDD systems. In CRT systems though, any attempt to lower their complexity and laborious implantation and thus their high complication rate should be welcome as long as CRT is properly implemented, as this would significantly lower medical care costs and the most important, patient prognosis. Many of these complications need entire system removal and replacement, which sometimes, especially in underdeveloped countries, is not possible because of the high costs. Patients usually have several comorbidities associated with their advanced heart failure, and many times a complication of the CRT system can be fatal. The idea of implementing cardiac resynchronization therapy through the simpler VDD CRT systems is gaining sight and this is proved by the fact that high-voltage single-pass VDD defibrillator leads for CRT are being developed. Data in the literature about these systems are scarce though and consist of very few reports and a very small number of patients. D’Ivernois et al. [10] predicting a lower complication rate for VDD CRT systems, reported chronic atrial sensing of 0.3–0.5 mV in a 7-year-old VDD lead and acute atrial sensing of 2.6 mV in a new one. The first system was upgraded from a simple to a VDD biventricular one, whilst the second one was a VDD biventricular to start with. The patients were followed up for a period of 34 and 24 months, respectively, and the authors reported biventricular pacing of 100% for both. The ejection fraction improved from 25% to 35% at 24 months and to 42% at 34 months in the first patient and from 22% to 27% in the second patient, without any system-related complications.

Gopi et al. [8], on the other hand, used the single-pass VDD lead placed in the coronary sinus, for right atrial sensing and left ventricle pacing and sensing in seven patients, reporting a proper efficiency and a higher availability for CRT implementation. After properly programming the AV interval to obtain fusion pacing with the physiological impulse coming from the RA through the AV node to depolarize the RV, they obtained a significant improvement of the LVEF after a six-month follow-up (from 25 ± 6% to 34 ± 6%). These results are similar to ours, and the patients had a similar age. The authors proved, even if on a small number of patients, that if the VDD lead is correctly placed, it can work properly, and it is safe and efficient for CRT even for extreme conditions of RA sensing-LV pacing in patients with advanced heart failure, severely dilated hearts, and severely impaired LVEF. There were no data though, on atrial sensing, and the follow-up was restricted to six months. Bellmann et al. [25] reported a proper functioning of the single-pass VDD defibrillator lead for CRT in two patients that were up-graded from simple VDD defibrillator systems to biventricular ones because of dilated cardiomyopathy development. Atrial sensing proved to be within very good limits (1.7 mV and 2.2 mV, and 1.2 mV and 3.0 mV, respectively, after four weeks), maintaining proper functioning of the CRT systems within the follow-up period, with a CRT biventricular pacing of 98% and 100%, respectively. Atrial rate histograms revealed normal activity. The authors concluded that the VDD CRT systems are feasible and efficient, with a less amount of foreign material.

Study limitation. We included a small number of patients in our study. Due to the nature of dilative cardiomyopathy, quantifying disease stage and life expectancy is difficult, and the severity of heart failure with which the patients are addressed for CRT therapy varies largely; thus, implantation protocols must be adapted to the individual patient. VDD CRT systems have not been extensively studied, and thus, they are not widely implanted. Because these systems were employed in patients with a specific anatomy (low subcutaneous tissue, particularities of the venous circulation, etc.) our study is not a randomized one, an aspect that represents another limitation of our study. Further, in this retrospective study, with a long-term follow-up period, several data were unavailable for a certain number of patients. For the same reason, certain data sets, such as the intervention/fluoroscopy time and other advanced CRT response criteria, were unavailable, so they were not included in the study.

## 5. Conclusions

VDD CRT systems may be a good alternative to the standardized DDD CRT systems, especially in patients with complicated anatomy that would not permit the implantation of a high number of leads. These systems tend to be safer and their performance seems to be similar to that of DDD CRT systems concerning CRT implementation and maintenance as well as patient evolution.

## Figures and Tables

**Table 1:** Patient and device-related characteristics at the moment of intervention.

	Group A 50 Patients	Group B 103 Patients	Group C 49 Patients	*p*
Age, mean ± SD, y	60.76 ± 10.22	61.76 ± 8.81	66 ± 9.21	0.045 ^a^
Gender, n (%)				
Male	39 (78%)	79 (76.69%)	33 (66%)	0.254 ^b^
Female	11 (22%)	24 (23.30%)	17 (34%)	
Etiology of DCM, n (%)				
Ischemic	9 (18%)	23 (22.33%)	15 (30%)	0.349 ^b^
Nonischemic	41 (82%)	80 (77.66%)	35 (70%)	
NYHA Class, n (%)				
Class II	6 (12%)	19 (18.44%)	26 (52%)	<0.001 ^b^
Class III	39 (78%)	65 (63.10%)	21 (42%)	
Class IV	5 (10%)	19 (18.44%)	3 (6%)	
LA, mean ± SD, cm	4.7 ± 0.38	4.88 ± 0.52	4.7 ± 0.53	0.035 ^a^
LVEF, mean ± SD, %	28.55 ± 7.21	26.89 ± 6.84	30 ± 5.45	<0.001^a^
Conduction delay, n (%)				
LBBB	40 (80%)	80 (77.66%)	47 (94%)	0.041 ^b^
Non-LBBB	10 (20%)	23 (22.33%)	3 (6%)	
QRS width, mean ± SD, ms	157.8 ± 23.23	159.61 ± 27.61	160 ± 19.08	0.825 ^a^
Diabetes mellitus, n (%)	8 (16%)	22 (21.35%)	24 (48%)	<0.001 ^b^
Chronic kidney disease, n (%)	19 (38%)	47 (45.63%)	25 (50%)	0.469 ^b^
Obesity n (%)	18 (36%)	24 (23.3%)	22 (44%)	0.026 ^b^
Medication				0.173 ^b^
ACE inhibitors, n (%)	18 (66.66%)	29 (61.70%)	25 (50%)	
B-Blokers, n (%)	20 (74.07%)	30 (63.82%)	44 (88%)	
Digoxin, n (%)	15 (55.55%)	22 (46.80%)	11 (22%)	
Mineralocorticoid receptor blocker, n (%)	22 (81.48%)	35 (74.46%)	27 (54%)	

Legend: *p*-statistical significance; DCM-dilated cardiomyopathy; n-number, NYHA-New York Heart Association; LA-left atrium; LVEF-left ventricular ejection fraction; LBBB-left branch bundle block; ACE- angiotensin converting enzyme; B-blocker-beta-blocker; SD-standard deviation; ^a^ Kruskal–Wallis test; post-hoc analysis with Mann–Whitney U test and Bonferroni correction; ^b^ Chi-square test.

**Table 2:** Complication rates in the three groups and their statistical significance.

	Group A	Group B	Group C
Complication	5 (10%)	24 (23.3%)	2 (4.1%)
No complication	45 (90%)	79 (76.7%)	47 (93.9%)

Legend: *p* = 0.003; Fisher exact test (Freeman–Halton extension).

**Table 3:** Functioning variables of the two systems in study subgroups.

	Subgroup A 27 Patients	Subgroup B 47 Patients	Subgroup C 36 Patients	*p*
Acute pacing/sensing thresholds				
Acute LV pacing threshold, mean ± SD, V	2.4 ± 1.73	1.97 ± 1.15	2.43 ± 0.79	0.25 ^a^
Acute LV sensing, mean ± SD, mV	12.21 ± 5.49	13.61 ± 5.27	10.82 ± 4.35	0.29 ^a^
Acute RV pacing threshold, mean ± SD, V	0.51 ± 0.15	0.54 ± 0.15	0.96 ± 0.57	0.63 ^a^
Acute RV sensing, mean ± SD, mV	15.90 ± 6.42	14.35 ± 4.64	12.12 ± 3.56	0.27 ^a^
Acute RA detection, mean ± SD, mV	3.31 ± 1.09	4.15 ± 3.08	3.32 ± 0.93	0.09 ^a^
Pacing differences between CRT typpes				
DDD pacing, n (%), mean ± SD				<0.001 ^b^
0%	27 (100%)	17 (36.17%)	36 (100)	
1–20%	0 (0%)	16 (34.04%)	0 (0%)	
21–41%	0 (0%)	8 (17.02%)	0 (0%)	
>40%	0 (0%)	6 (12.76%)	0 (0%)	
VVI pacing, %,,mean ± SD	1.7 ± 2.24	0 (0%)	0(0%)	<0.001 ^a^
VVI pacing, n (%)				<0.001 ^b^
0%	15 (53.57%)	77 (100%)	36 (100)	
1–5%	10 (39.28%)	0 (0%)	0 (0%)	
6–10&	2 (7.14%)	0 (0%)	0 (0%)	
Functioning variables of the two systems				
CRT pacing, mean ± SD, %	98.31 ± 2.65	98,02 ± 3.3	98.18 ± 1.64	0.89 ^a^
CRT pacing, n (%)				0.41 ^b^
≥98%	21 (77.77%)	36 (76.59%)	36 (73.46%)	
<98%	6 (22.22%)	11 (23.40%)	13 (26.53%)	
93–98%	5 (21.42%)	8 (17.02%)	12 (24.48%)	
<93%	1 (3.57%)	3 (6.38%)	1 (2.04%)	
Ventricular detection > 2%, n (%)	3 (11.11%)	6 (12.76%)	0 (0%)	1 ^b^

Legend: LV-left ventricle; SD-standard deviation; RV-right ventricle; RA-right atrium; VVI-ventricular sensing and pacing; CRT-cardiac resynchronization therapy; ^a^ Kruskal–Wallis test; post-hoc analysis with Mann–Whitney U test and Bonferroni correction; ^b^ Chi-square test.

## Data Availability

The data are available at doi:10.17632/ns68d3nnc2.1, accessed on 16 July 2021.

## References

[1] Brignole M., Auricchio A., Baron-Esquivias G., Bordachar P., Boriani G., Breithardt O.-A., Cleland J., Deharo J.-C., Delgado V. (2013). ESC Guidelines on Cardiac Pacing and Cardiac Resynchronization Therapy: The Task Force on Cardiac Pacing and Resynchronization Therapy of the European Society of Cardiology (ESC). Developed in Collaboration with the European Heart Rhythm Association (EHRA). Europace.

[2] Lawin D., Stellbrink C. (2019). Change in Indication for Cardiac Resynchronization Therapy?. Eur. J. Cardiothorac. Surg..

[3] Normand C., Linde C., Blomström-Lundqvist C., Stellbrink C., Gasparini M., Anker S.D., Plummer C., Sarigul N.U., Papiashvili G., Iovev S. (2020). Adherence to ESC Cardiac Resynchronization Therapy Guidelines: Findings from the ESC CRT Survey II. EP Eur..

[4] Epstein A.E., DiMarco J.P., Ellenbogen K.A., Estes N.A.M., Freedman R.A., Gettes L.S., Gillinov A.M., Gregoratos G., Hammill S.C., Hayes D.L. (2008). ACC/AHA/HRS 2008 Guidelines for Device-Based Therapy of Cardiac Rhythm Abnormalities. J. Am. Coll. Cardiol..

[5] Margey R., McCann H., Blake G., Keelan E., Galvin J., Lynch M., Mahon N., Sugrue D., O’Neill J. (2010). Contemporary Management of and Outcomes from Cardiac Device Related Infections. Europace.

[6] Poole J.E., Gleva M.J., Mela T., Chung M.K., Uslan D.Z., Borge R., Gottipaty V., Shinn T., Dan D., Feldman L.A. (2010). Complication Rates Associated With Pacemaker or Implantable Cardioverter-Defibrillator Generator Replacements and Upgrade Procedures: Results From the REPLACE Registry. Circulation.

[7] Kirkfeldt R.E., Johansen J.B., Nohr E.A., Moller M., Arnsbo P., Nielsen J.C. (2011). Risk Factors for Lead Complications in Cardiac Pacing: A Population-Based Cohort Study of 28,860 Danish Patients. Heart Rhythm.

[8] Gopi A., Sundar G., Yelagudri S., Lalukota K., Sridevi C., Narasimhan C. (2014). Atrial Synchronous Left Ventricular Only Pacing with VDD Pacemaker System–A Cost Effective Alternative to Conventional Cardiac Resynchronization Therapy. Indian Heart J..

[9] Boriani G., Berti E., Belotti L.M.B., Biffi M., Carboni A., Bandini A., Casali E., Tomasi C., Toselli T., Baraldi P. (2014). Cardiac Resynchronization Therapy: Implant Rates, Temporal Trends and Relationships with Heart Failure Epidemiology. J. Cardiovasc. Med..

[10] D’Ivernois C., Pi S., Hero M. (2005). Cardiac Resynchronization Therapy Using a VDD Lead. Pacing Clin. Electrophysiol..

[11] Wiegand U.K.H. (2012). Single-Lead VDD Pacing--a Serious Alternative for Atrioventricular Synchronous Pacing in Patients with Atrioventricular Block?. Europace.

[12] Schaer B.A., Weinbacher M., Zellweger M.J., Sticherling C., Osswald S. (2007). Value of VDD-Pacing Systems in Patients with Atrioventricular Block: Experience over a Decade. Int. J. Cardiol..

[13] Blich M., Suleiman M., Shwiri T.Z., Marai I., Boulos M., Amikam S. (2010). Long-Term Outcome of Atrial Synchronous Mode Pacing in Patients With Atrioventricular Block Using a Single Lead. Clin. Cardiol..

[14] Huang C.-C., Tuan T.-C., Fong M.-C., Lee W.-S., Kong C.-W. (2010). Predictors of Inappropriate Atrial Sensing in Long-Term VDD-Pacing Systems. Europace.

[15] Hayes D.L., Boehmer J.P., Day J.D., Gilliam F.R., Heidenreich P.A., Seth M., Jones P.W., Saxon L.A. (2011). Cardiac Resynchronization Therapy and the Relationship of Percent Biventricular Pacing to Symptoms and Survival. Heart Rhythm.

[16] Koplan B.A., Kaplan A.J., Weiner S., Jones P.W., Seth M., Christman S.A. (2009). Heart Failure Decompensation and All-Cause Mortality in Relation to Percent Biventricular Pacing in Patients With Heart Failure. J. Am. Coll. Cardiol..

[17] Tracy C.M., Epstein A.E., Darbar D., DiMarco J.P., Dunbar S.B., Mark Estes N.A., Ferguson T.B., Hammill S.C., Karasik P.E., Link M.S. (2012). 2012 ACCF/AHA/HRS Focused Update of the 2008 Guidelines for Device-Based Therapy of Cardiac Rhythm Abnormalities. J. Thorac. Cardiovasc. Surg..

[18] Normand C., Linde C., Singh J., Dickstein K. (2018). Indications for Cardiac Resynchronization Therapy. JACC Heart Fail..

[19] Lakkireddy D.R., Chung M.K., Gopinathannair R., Patton K.K., Gluckman T.J., Turagam M., Cheung J.W., Patel P., Sotomonte J., Lampert R. (2020). Guidance for Cardiac Electrophysiology during the COVID-19 Pandemic from the Heart Rhythm Society COVID-19 Task Force; Electrophysiology Section of the American College of Cardiology; and the Electrocardiography and Arrhythmias Committee of the Council on Clinical Cardiology, American Heart Association. Heart Rhythm.

[20] (2016). ESC Guidelines for the Diagnosis and Treatment of Acute and Chronic Heart Failure. Eur. Heart J..

[21] Shurrab M., Elitzur Y., Healey J.S., Gula L., Kaoutskaia A., Israel C., Lau L., Crystal E. (2014). VDD vs DDD Pacemakers: A Meta-analysis. Can. J. Cardiol..

[22] Marchandise S., Scavee C., le Polain de Waroux J.-B., de Meester C., Vanoverschelde J.-L., Debbas N. (2012). Long-Term Follow-up of DDD and VDD Pacing: A Prospective Non-Randomized Single-Centre Comparison of Patients with Symptomatic Atrioventricular Block. Europace.

[23] Chugh S.S., Ellenbogen K.A., Wilkoff B.L., Kay G.N., Lau C.-P., Auricchio A. (2017). Clinical Cardiac Pacing, Defibrillation, and Resynchronization Therapy.

[24] Al-Majed N.S., McAlister F.A., Bakal J.A., Ezekowitz J.A. (2011). Meta-Analysis: Cardiac Resynchronization Therapy for Patients With Less Symptomatic Heart Failure. Ann. Intern. Med..

[25] Bellmann B., Tscholl V., Landmesser U., Roser M. (2015). Kardiale Resynchronisation mit VDD-Elektrode: Geht das?. Herzschrittmachertherapie Elektrophysiologie.

